# A Qualitative Assessment of Place and Mental Health: Perspectives of Young Women Ages 18–24 Living in the Urban Slums of Kampala, Uganda

**DOI:** 10.3390/ijerph191912935

**Published:** 2022-10-10

**Authors:** Monica H. Swahn, Jacqueline Nassaka, Anna Nabulya, Jane Palmier, Seneca Vaught

**Affiliations:** 1 Health Promotion and Physical Education, Wellstar College of Health and Human Services, Kennesaw State University, Kennesaw, GA 30144, USA; 2 Uganda Youth Development Link, Kampala P.O. Box 12659, Uganda; 3 Wellstar College of Health and Human Services, Kennesaw State University, Kennesaw, GA 30144, USA; 4 Interdisciplinary Studies, Radow College of Humanities and Social Sciences, Kennesaw State University, Kennesaw, GA 30144, USA

**Keywords:** place, mental health, slums, Africa, Uganda, participatory research, photography

## Abstract

This paper examines the link between place and mental health using a qualitative assessment and focus group discussion with young women, ages 18 to 24 years of age, residing in three slums in Kampala, Uganda. The assessment, conducted in August of 2022, engaged 15 women who participated in Uganda Youth Development Drop-in center activities. The objective was to assess mental health and the link between place and mental health. Facilitated group discussions and photograph review yielded the following results. In terms of understanding their views of mental health and wellbeing, participants clearly focused on feelings. However, they also assessed resilience, the environment and a person’s choice as relating to their mental health. Participants also found the physical spaces related to sports, education, worship, workplaces and green space to be linked to happiness. In terms of the attributes that were linked to sadness, participants listed the physical locations where drugs are sold, clubs for dancing and partying and also sanitation issues in the community. Participants frequently reported on the social environment and reflected on harassment, discrimination, alcohol use and criminal behavior that did not reflect a specific physical space, but rather the embedded social interactions they may face or observe by living in close proximity to hotspots for criminal activity. Given the dire shortages of mental health services and care that are available in this setting, a better understanding of young women’s perceptions of place and mental health will be key for low-cost interventions and strategies to mitigate the contextual factors that may exacerbate mental illness.

## 1. Introduction

It has been long recognized that place impacts health [1,2,3]. The Centers for Disease Control and Prevention (“CDC”) for example, states that “the places of our lives—our homes, workplaces, schools, parks, and houses of worship—affect the quality of our health and influence our experience with disease and well-being” [1]. In their conceptual approach, the CDC outlines several features of “place”, and that the natural environment, the built environment, the population connectivity environment, the social and behavioral environment and health policy environment all interact in many ways to contribute to health in complex ways. This is a challenging area of public health research that has seen a tremendous interest and growth [4,5,6]. In particular, the complexity of establishing empirical linkages between place and health outcomes has been well recognized, with calls for how the impacts of place can be conceptualized, operationalized and measured [7]. However, specific research on the impact of place among vulnerable populations in urban slums has been much scarcer, despite the fact that slums are now home to over 1 billion people [8,9].

Urban slums are rapidly growing, primarily due to population growth and rural migration, and it is estimated that 80% of those who live in urban slums can be found in Eastern and South- Eastern Asia (370 million), Sub-Saharan Africa (238 million) and Central and Southern Asia (227 million) [9]. New data from the UN-Habitat show that most urbanization will occur in low-income countries, with projected growth of 56% in 2021 to 68% in 2050, translating into an increase of 2.2 billion urban residents, living mainly in Africa and Asia [8]. Across Sub-Saharan Africa, over 60% of the urban population live in slums [10]. In Uganda, one of the countries with the highest population growth [11,12], about half of the population live in slums [13]. Slums are particularly important in the form of “place”, as the very definition of the word slum indicates hardship, e.g., “*a densely populated usually urban area marked especially by poverty*” [14]; similarly, a slum can be defined as “*a residential area with substandard housing that is poorly serviced and/or overcrowded, and therefore unhealthy, unsafe, and socially undesirable*” [15]. Informal human settlements, another term also used to describe slums, describes these settings as having low-quality housing, overcrowding, pollution, limited basic infrastructure, and that they comprise unsafe residential conditions [16]. These descriptions indicate the close linkages of slums or informal human settlements with the concept of social determinants of health (SDoH). While there are many definitions, typically the SDoH refer to the non-medical factors that influence health outcomes and typically focus on income, education, employment, food security, housing and similar factors.

Research on the social determinants of health and unmet health needs among slum dwellers or more succinctly “slum health” is emerging and gaining more interest, as the proportion of slum dwellers are rapidly increasing across multiple continents as part of rural migration [17,18,19]. One study noted that those living in slums represent a great concern, as “*this neglected population has become a major reservoir for a wide spectrum of health conditions that the formal health sector must deal with*” [20]. There are many complexities embedded in studying slums and arguably their study needs to be contextualized to represent both social and physical constructs [21]. Also, as the slum environment is embedded in the context of the SDoH and furthermore contextualized by the strong link between poverty and mental health, it is important to better determine the specific factors that may exacerbate mental health among those living in the slums.

The qualitative assessment for this study is part of a broader project with several embedded research studies which we refer to as The Onward Project On Well-being and Adversity (“TOPOWA”) study (meaning empowerment to keep pushing forward and never giving up, in Luganda, the local language spoken in Kampala). This study will examine the mechanisms by which socioeconomic strengthening-targeted training moderates the pathways between the adverse effects of poverty (proximal social and environmental stressors) and mental illness among urban youth in Kampala, and builds on the social determinants of mental health (SDoMH) [22,23,24,25] and the research domain criteria (RDoC) [26,27] frameworks. We propose in our NIH-funded study that social and environmental stressors may act independently or through interaction with interpersonal characteristics to exacerbate mental illness in the context of urban poverty, a topic that remains poorly understood [23,28,29,30]. Our extensive previous research of urban youth, including adolescent girls and young women in Kampala, demonstrates high levels of distress, loneliness, sadness and suicidal ideation. These feelings are typically in in response to adverse childhood experiences and adversities including economic instability, food and shelter insecurity, and homelessness, in addition to domestic and community violence, sexual exploitation and physical abuse, family separation and parental death [31,32,33,34,35,36,37,38,39,40,41,42,43,44,45,46,47,48,49,50,51]. However, the lack of comprehensive research on interventions and factors that may buffer against the relationships between poverty, other environmental stressors and mental illness is a significant barrier to progress.

Within the Ugandan context, conceptualizing and contextualizing mental health for young women remains an important priority. Mental health is often portrayed as an abstract concept, focusing primarily on the most severe of mental disorders that are also associated with significant stigma. As such, there are limited opportunities for understanding the barriers women face and the opportunities for intervention and prevention. A key goal of our TOPOWA study is to capture the real-world complexity of the urban slums and to deliberately assess neighborhood characteristics, the women’s activity space and their views of mental illness. As such, the SDoMH framework is central to this study of young women who live in the slums of Kampala and clearly recognizes the impact of the environment and neighborhood effects; therefore, we will also examine adverse neighborhood characteristics that are likely to exacerbate stress, substance use and mental illness. A growing body of literature, new geospatial methods, and tools have been developed to link place to mental health [52]. While the theoretical underpinning of this work is still evolving, tools such as the Neighborhood Inventory for Environmental Typology (NIfETy) method [53] for conducting environmental assessment of neighborhood-level indicators of violence, alcohol and other drug exposure will be helpful in assessing the proximal environmental stressors experienced by young women in urban slums [54,55]. Research indicates that the NIfETy tool is valid and economical for assessing neighborhood characteristics in the U.S. [56]. However, few if any similar environmental typology tools exist to examine or assess indicators in global low-resource settings, or in slums specifically [57].

In the U.S., much of the research on the urban socioenvironmental context and mental illness has focused on the alcohol environment specifically [58,59,60]. Although the specific mechanisms are unclear, research has found that U.S. women who live in alcohol-dense neighborhoods are about twice as likely to report depression than women living in less alcohol-dense environments [60]. Studies on these or similar neighborhood indicators have rarely been conducted in Sub-Saharan Africa. Accordingly, we have made it a priority as part of our TOPOWA study to map the neighborhood level indicators (e.g., alcohol outlets, gambling facilities, pleasure halls) in the communities where study participants in the future prospective cohort reside. However, it will be critically important to first determine how young women see the link between place and mental health. More specifically, we need to know what factors they see in terms of place and environment that serve as stressors and impact mental health in their own settings. To our knowledge, this type of assessment has not been conducted previously, but is a key step in making progress on better understanding the link between place and mental health.

The information presented in this paper is based on our formative research through the focus groups discussions that are embedded in a participatory photography project using the PhotoVoice approach [61,62]. The goal of this project component is to give us insight into the specific neighborhood characteristics perceived to be most relevant by young women, and potentially linked to mental health outcomes, in their own views. Additionally, the PhotoVoice project will be instrumental in understanding young women’s proximal social and environmental stressors within their own neighborhoods, as participants will be instructed to reflect on the social and physical constructs of neighborhood characteristics in the slums [21] and how these may intersect to exacerbate mental illness. In this qualitative assessment, we engage with 15 young women, 18 to 24 years of age, to determine how they view their urban environment and what features of the environment may contribute to the condition of their mental health. Findings from this formative research will guide the future research components, including survey assessments and environmental neighborhood indicator mapping tools, and can also serve as a strategic approach for elucidating these concepts for better assessment in future research.

## 2. Methods

This qualitative assessment was completed with 15 young women, 18 to 24 years of age, on 17 August 2022. Participants were recruited from three youth support centers operated by the Uganda Youth Development Link across metropolitan Kampala, Uganda. Participants were informed that researchers from Kennesaw State University, Kennesaw, GA, USA and Makerere University, Kampala, Uganda are doing a study entitled TOPOWA (The Onward Project on Wellbeing and Adversity) to learn about young women and their community and how their environment impacts their well-being and mental health.

The qualitative assessment presented in this paper is a study component of a broader participatory photography project using the PhotoVoice approach [62,63]. In this case, the PhotoVoice methodology uses photography as a way for young women to tell their own story about their community and not giving up. TOPOWA means not to give up in the local language, Luganda. This project necessitated an in-depth training and focus group-like discussion with the women to better understand how young women see mental health and how mental health may be impacted by certain and specific features of the urban slums. This report, therefore, gives details of the one-day TOPOWA PhotoVoice training and the focus group-type discussions which were embedded as part of the training, representing a census of the responses by all participants.

We obtained ethical approval in 2022 from the Makerere University School of Social Sciences Research Ethics Committee (*MAKSSREC 06.22.566*) and the Uganda National Council of Science and Technology (*registration number SS1347ES*). Written informed consent was obtained from all study participants before data collection. Participants were selected from the Uganda Youth Development Link (UYDEL) drop-in centers for vulnerable youth. UYDEL is a non-governmental organization that works with youth ages 10–24 to provide psychosocial support, services, and skills training to vulnerable youth. They operate drop-in centers as well as a larger residential rehabilitation center. For this project, we selected three different centers for participant recruitment, the Makindye Youth Centre, the Bwaise Youth Centre and the Banda Youth Centre, all in the Kampala district.

The training was led by two women facilitators, representing the leadership at UYDEL. Both facilitators were CITI-trained and -certified and were also trained in the methodology to be used for the PhotoVoice and focus group engagement with participants. One of the facilitators had also received specific training in participatory photography and had previous experience with research projects. The project’s Principal Investigator (Dr. Swahn) has also been trained in the PhotoVoice approach. English is the official language in Uganda. However, the facilitators are fluent in both English and Luganda (local language) and could facilitate translation as needed for participants who may have expressed some statements in the local language.

The fifteen participants ranged in age from 18 to 24 years of age. None of the women were married, but one was cohabiting with a partner. Only one of the participants received upper secondary schooling; the remaining had obtained education at lower secondary (9 women) and primary school (5 women). In terms of religion, nine identified as Christian and six identified as Muslim. Only two of the participants were mothers and had one child each. More than half (8) of the participants were unemployed, four were self-employed and two were employed. Among those who were employed, the income earned per month ranged from 15,000–320,000 Uganda Shillings (which converted to dollars would be about $4.30–91.42). Overall, their family size ranged from one to ten people. The top two employment skills listed were hairdressing/cosmetology (8 women) and bakery (3 women).

### Data Collection

The embedded training and focus group-type discussions included three data collection components that raised the following questions for a facilitator-led group discussion:

1. What is mental health and wellbeing? What does it mean to you? (Focus group discussion)
2. Is there a link between place, mental health and wellbeing? (Focus group discussion)
  - What makes you happy in your community?
  - What makes you feel sad in your community?
3. What specific aspects in your community reflect feelings of happy, sad, or stressed. (A review of 25 photos).

These new exercises and data collection strategies were developed by Dr. Swahn in consultation with the research team to meet the overarching project objectives of understanding the link between place and mental health. The resident photographer furnished the photos to represent various setting and aspects of the built and social environment to facilitate conversations on place, mental health and wellbeing. Per directions from the study team, the resident photographer was asked to capture the built environment and different physical and social scenes, reflecting aspects of the slums that we anticipated would be linked to mental health (e.g., latrines and sanitation, water drainage, trenches, bridges for sewage crossings, markets, residences, places of entertainment and worship, alcohol marketing and sales). The final 25 photos were selected for participant review in consultation with the study team to reflect diverse and typical settings within the slums. The photos were selected also to reflect the three centers from which participants were recruited. Also, because the young women often have a hard time articulating aspects and the range of mental health concerns (per facilitators), we opted to specify three specific feelings: happiness, sadness, and stress. Providing and anchoring the conversations regarding mental health in this way operationalized an otherwise abstract concept that is not often discussed within the cultural context.

Responses to the focus group-like discussions were transcribed and summarized into themes for each of the three different topics. Themes were interpreted and ideas were grouped within the overarching context. Any responses provided in Luganda were translated into English. All responses from the 15 participants were included in the analyses. Because of the few respondents, no software was needed to identify themes. While participants were encouraged to discuss the emotional reactions to the photographs, there were no specific discussions of pictures that did not elicit any emotions.

## 3. Results

### 3.1. What Is Mental Health and Wellbeing? What Does It Mean to You?

Participants’ Responses to “What is mental health & wellbeing? What does it mean to you?” elicited 20 different statements. Most of the responses comprised statements about feelings, what they felt, whether positive or negative, such as “*Mental health is how you feel either bad or good*”, *Emotional feelings either bad or good*”, “*Things that disturb our minds and affect how we feel*”, “*Feelings of the person on anything that affects a person on how they feel*”, “*Concerns feelings like being moody or happy*”, *Psychological wellbeing either positive or bad*” or “*The way we feel and behave*”.

Some also responded with specific mental health concerns such as “*Anxiety or Depression*” “*Things that stress me*” or referenced the brain “*Concerning my brain and emotions*” or treatment as in “*Aspect of mental care*”. Some responses were broader, such as statements reflecting “*Emotions and behavior*”.

Some participants also distinguished between physical and psychological health in the following way “*Physical when touched and psychological when not touched*”. With respect to wellbeing, participants found that it could be “*social and psychological*” and “*Social or emotional wellbeing*”.

Several participants also referenced the environment or community as a response to feelings, saying “*Make the environment happy*”, and also that mental health reflected choices, as in “*How you move or live your life*”, “*The way we respond to situations we face in life*”, or how people act “*Proper behavior in the community*”, or having capacity and resilience “*Have a strong mind that can hold any situation*”.

### 3.2. Is There a Link between Place, Mental Health and Wellbeing? What Makes You Happy in Your Community?

Participants were also asked to think about the link between place and mental health and, more specifically, what makes them happy or sad when they are in their communities.

In terms of places that reflected happy mood, participants mentioned a few specific places such as “*The football pitch in my community—for my love for sports*”, “*The green garden around the school next to my home area that is well decorated makes me happy and I feel peaceful*”, “*There is a Club near my home and it makes me happy because I like listening to positive music*”, “*Local Cinema halls are near and they don’t discriminate that it’s for only boys but also girls can access them too and this is very entertaining*”, and “*My center UYDEL in Bwaise makes me happy. It has been able to support all the youth in the community and I have created a new social support network with the staff and fellow youth*”.

Participants also mentioned places of worship such as “*Church accessibility—it’s near my home so I am able to connect with my God*”, and “*There is a Mosque near my home, I am able to connect with Allah on a daily basis*”. Participants also mentioned the schools, “*There are many Schools in my community, which is a positive thing as it shows that children can access education and learn*” and “*Police stations that are able to put control measures for criminals thus security in my community*”. One participant mentioned “*Accessibility to retail shops*”.

### 3.3. Is There a Link between Place, Mental Health and Wellbeing? What Makes You Sad in Your Community?

In terms of places that make participants sad, they mentioned “*Drugs selling hotspots*”, and “*The nearest club with loud music makes me have sleeping disorders*”. Several participants also mentioned sanitation concerns such as “*Poor disposal of rubbish by the people in my community*” and specific places such as “*The toilet at home got full and my family is not able to dig another one, so we are forced to share with the neighbor. It is difficult to get the toilet key from the neighbor as she always demands that we first fetch for her water before we can access her toilet which is disturbing especially when you are in urgent need of using the bathroom so you need to prepare early or avoid over eating and drinking to avoid using the toilet many times*” and “*Sanitation is not good, whenever it rains, the rubbish in the drainage system all gathers next to my house and I have to clean it up to avoid diseases and flooding*”. However, more of the discussion reflected disrespect and harassment from other people in their community, rather than specific places, such as “*The many idle youth who disrespect us, especially the girls in my community*”, “*Finding fellow youth talking about other people*”, “*Finding someone who has taken alcohol and uses vulgar words*”, “*Bad touches from Boda boda men*”, and “*Indecent wear from the girls from the bars in the community*”.

Other negative interactions among people in the community reflected lack of collaborations, dishonesty and crime as in comments such as the following, which described “*Unfriendly colleagues who don’t like to collaborate with others*”, “*Dishonest people in the community, who keep lying and hood winking others*”, and “*Crimes in the community are very many mostly committed by those youth that abuse alcohol and drugs*”, “*There are very many idle people in the community, who end up stealing and committing crimes*”. One comment was also made regarding smoking in public “*Finding someone smoking cigarettes in public*”. Finally, one comment was made about lack of access to health care, “*No free medical services because we don’t have a big hospital that can help during emergencies or provide free medication and treatment*”.

### 3.4. Photograph Review

**Figure 1:**
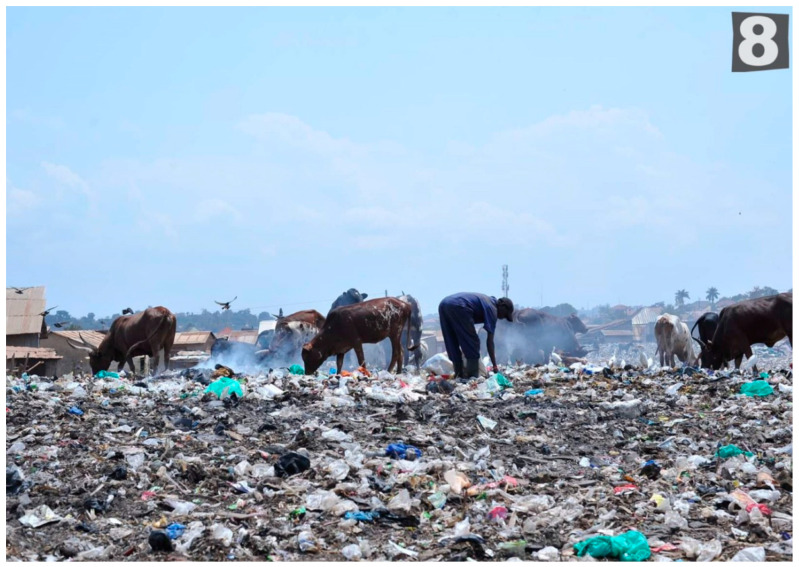
Garbage and Livestock (photo number 8).

**Figure 2:**
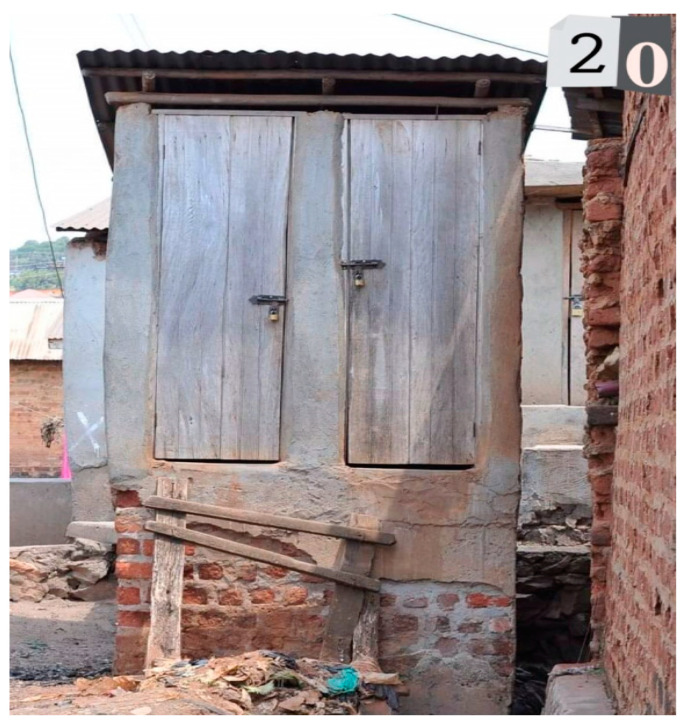
Community Latrine (photo number 20).

**Figure 3:**
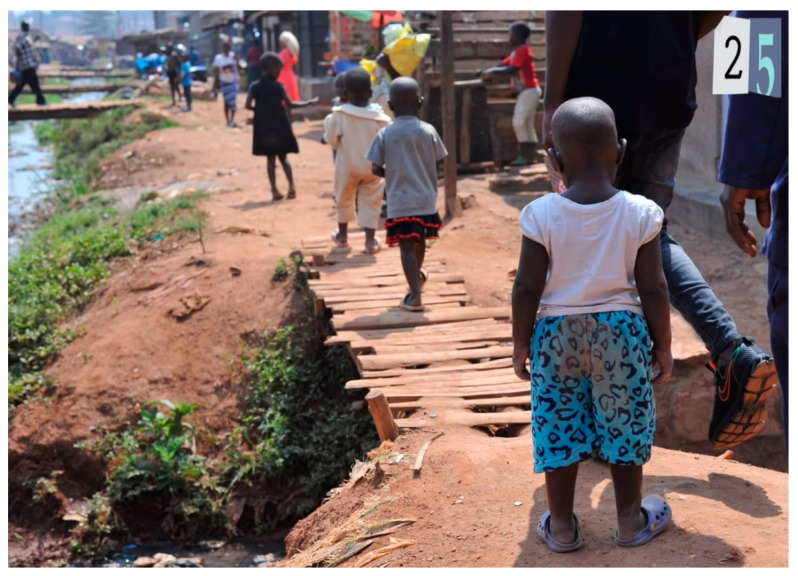
Children Walking over Bridge (photo number 25).

In the third task—a participatory review of photographs to indicate which may reflect places and mental health and emotions such as happiness, sadness and stress—participants engaged in three small groups and discussed with the facilitator what photos may reflect for each participant or what multiple emotional responses were experienced (Table 1). Of the 25 photos, 9 reflected happy, 16 reflected sad and 18 reflected stressed emotions. Half of the photos (12) reflected both sad and stressed and depicted a bar, a church, garbage and livestock (Figure 1), crowded slum, people walking next to a trench, a home, latrine (Figure 2), women entrepreneurs cooking, alcohol marketing, a boda-boda stand, and children walking over bridge (Figure 3). A few pictures are included to represent these feelings.

**Figure 4:**
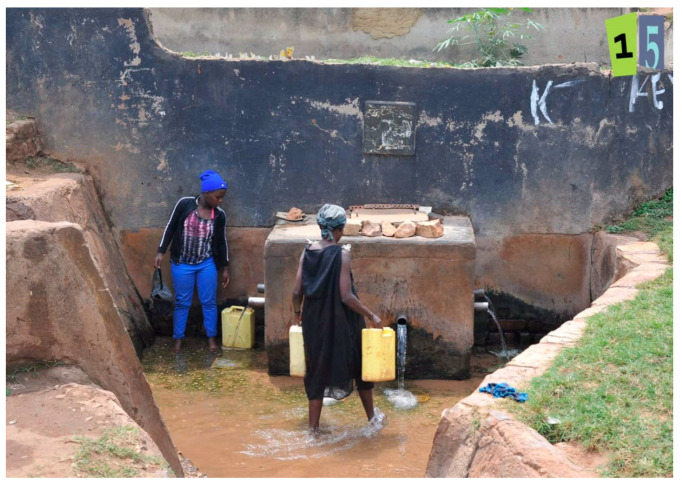
Women Fetching Water in a Well (photo number 15).

Four photos reflected multiple emotional responses of happy and sad, and depicted the weekly market, women entrepreneurs cooking, women fetching water in a well (Figure 4), and a movie hall for entertainment.

**Figure 5:**
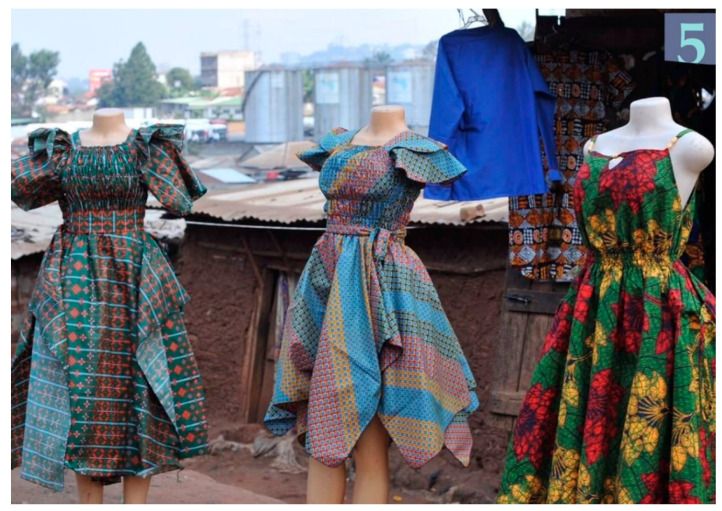
3 African Print Dresses (photo number 15).

**Figure 6:**
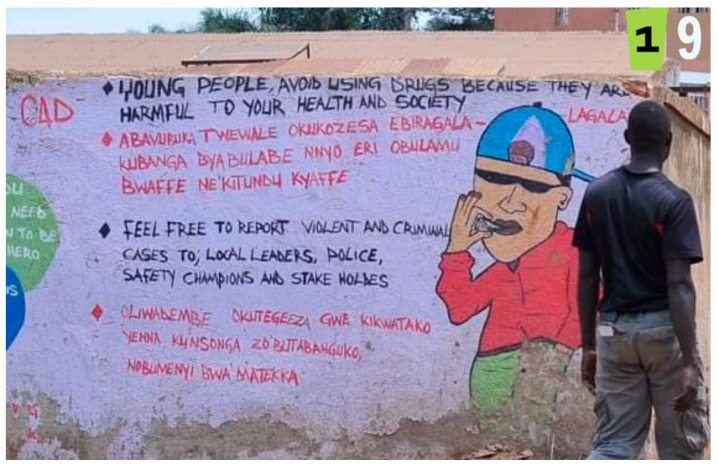
Mural Prevention Message (photo number 19).

Four photos specifically elicited a response of happy and depicted a scene with three African print dresses (Figure 5), a mosque, a medical clinic, and a photo of a prevention message on a mural wall (Figure 6).

**Figure 7:**
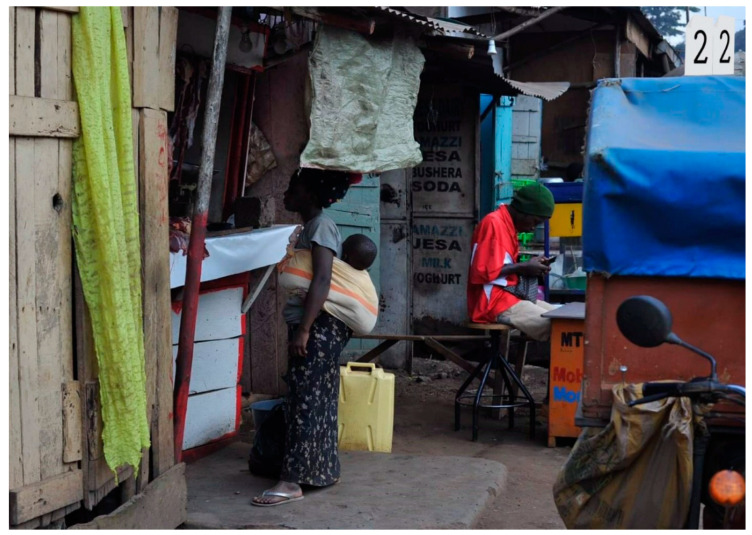
Mother With Child Shopping (photo number 22).

One photo depicted all three emotions and reflected a young woman with child shopping for food (Figure 7).

## 4. Discussion

In this formative research and qualitative explorative assessment using new and innovative strategies, we examined the aspects of mental health, as well as the link between mental health and the social and proximal physical attributes of the urban slums, as reported by young women ages 18 to 24, in Kampala, Uganda. We found important themes that can be incorporated into future research, participatory action projects and also community assessment to better understand the intersection between the slum environment and emotional experiences of slum residents.

In terms of understanding their views of mental health and wellbeing, participants clearly focused on feelings, as expected. However, they also attributed resilience, the environment and a person’s choice as relating to their mental health. While we did not probe deeper on this topic, nor assessed participant’s own mental health or wellbeing, these themes raise key questions about agency and embodiment worth further exploration.

Our findings show that the participants find several and specific physical spaces related to sports (football pitch), education (schools), worship (churches and mosques) and green space (gardens) linked to happiness which mirrors previous research in other settings [5,57]. Participants specifically referenced these structures in their narratives in terms of accessibility, social support and community which are important factors as we consider the link between poverty and mental illness and how the urban slums typically lack some of these structures which may create a sense of isolation despite the typically high population density [8,64].

In terms of the attributes that were linked to sadness, participants listed specific physical locations where drugs are sold, clubs for dancing and partying and also sanitation issues. Sadly, participants frequently reflected on the social environment by mentioning concerns related to harassment, discrimination, alcohol use, and criminal behavior that did not reflect a specific physical space, but rather the embedded social interactions they may face or observe by living in close proximity to hotspots for criminal activity [21].

Additionally, a couple of comments also reflected the appreciation for inclusion. One woman commented regarding the “*local cinema halls are near, and they don’t discriminate that it’s only for boys but also girls can access them too and it is very entertaining*”. This statement hints at differing opportunities for entertainment for young men and women in the community, but that these cinema halls (basic structures with a movie screen or TV) are more inclusive and can be enjoyed by both men and women.

Other key findings included the many references to poor sanitation and its impact on both individuals and the community. In terms of understanding place and health, a key focus in urban slums has been specifically on sanitation as the lack of infrastructure in these informal settlements directly impacts the accumulation of garbage and wastewater. There is a growing interest in understanding the health impacts of sanitation interventions and hygiene [65,66], usage of latrines [67], open defecation free status [68] and also menstrual hygiene [69] in low-resource settings. Menstrual hygiene was not mentioned in the current project, but likely directly impacts participants. However, the link between sanitation concerns such as use of pit latrines, their safety and mental health needs further exploration.

Finally, the findings from our review of photographs to examine the physical attributes of the urban slums underscore the complexity of disentangling the physical and social environments. Participants highlighted a few key physical attributes that represented happiness in some ways. They indicated that the depiction of dresses for sale, a mosque, a medical clinic and a painted wall mural with a prevention message all represented happiness, and happiness only. That is unlike many of the other photographs which reflected both sadness and stress, while a few reflected only sadness.

This study is an explorative qualitative assessment of the link between place and mental health in a group of 15 women who live in three urban slums in Kampala. The thematic findings from this qualitative assessment are meant to provide a voice and insight from participants who rarely have a chance to share their views and perspectives on their lived experience, hardships and physical environment. The limitations inherent in our qualitative approach is that the findings may not generalize to other populations and to other settings. Moreover, our approach was exploratory given the dearth of research and tools available to assess the link between place and mental health in this and in similar populations in urban slums. We could not find a similar project. However, our future project plans required us to apply an innovative approach for eliciting responses and for engaging the young women to think about their environments and the physical and social attributes that make a difference in their lives and how they feel, whether they are happy, stressed, or sad. A limitation (and a strength) of our exploratory approach is that we also truncated potential responses to the specific feelings. We used these to anchor responses. Future approaches may want to consider a broader range of emotions and mental health concerns to probe deeper into the contextual aspects of place and mental health. It may also be worthwhile to specifically ask about those attributes that do not elicit any emotional response.

## 5. Conclusions

While many of the findings obtained in this assessment were expected, we also found that the insight provided by these women provide important directions for our future research, particularly with respect to the intertwining social and physical attributes of the environment, the mixed reactions to places of worship, the reports of harassment, discrimination and crime, and also the many adverse impacts related to poor sanitation. In addition, these findings can further how reflective communal inquiry and the emotional experiences of women in the slums are factored into urban contextual planning. Given the dire shortages of mental health services and care that are available in this setting, a better understanding of women’s perceptions of place and mental health will be key for low-cost interventions and strategies to mitigate the contextual factors that exacerbate hardships and mental illness.

## Figures and Tables

**Table 1:** Participants’ Group Response to 25 Photos of Places within Urban Slums that are Linked to Mental Health and Elicit an Emotional Response.

Photo #	Description	HAPPY	SAD	STRESSED
Photo 1	VIP Bar & Inn		√	√
Photo 2	Train Tracks			√
Photo 3	Blue Church		√	√
Photo 4	Business Area		√	
Photo 5	3 African Print Dresses	√		
Photo 6	Plastics		√	
Photo 7	Weekly Market	√		√
Photo 8	Garbage and Livestock		√	√
Photo 9	Women Entrepreneurs Cooking	√		√
Photo 10	Crowded Slum		√	√
Photo 11	Mosque	√		
Photo 12	Busy Day-to-Day (gas tank)		√	√
Photo 13	Woman Fetching Dirty Water		√	
Photo 14	Movie Hall	√		√
Photo 15	Women Fetching Water in Well	√		√
Photo 16	Medical Clinic	√		
Photo 17	People Walking Next to Trench		√	√
Photo 18	A Home		√	√
Photo 19	Mural Prevention Message	√		
Photo 20	Community Latrine		√	√
Photo 21	Women Entrepreneurs Cooking		√	√
Photo 22	Mother with Child Shopping	√	√	√
Photo 23	Alcohol Marketing (Senator)		√	√
Photo 24	Boda-Boda Stand		√	√
Photo 25	Children Walking w Bridge		√	√

## Data Availability

The data presented in this study are available on request from the corresponding author.
